# Green Strong Cornstalk Rind-Based Cellulose-PVA Aerogel for Oil Adsorption and Thermal Insulation

**DOI:** 10.3390/polym16091260

**Published:** 2024-05-01

**Authors:** Xiaoyang Yi, Zhongxu Zhang, Junfeng Niu, Hongyan Wang, Tiankun Li, Junjie Gong, Rongbo Zheng

**Affiliations:** 1 College of Materials and Chemical Engineering, Southwest Forestry University, Kunming 650224, China; xiaoyang_yi@163.com (X.Y.); zzy@swfu.edu.cn (Z.Z.); 2 College of Biological & Chemical Engineering, Zhejiang University of Science and Technology, Hangzhou 310023, China; 103044@zust.edu.cn (J.N.); qbh040915@163.com (T.L.); 15224094790@163.com (J.G.); 3 Academy of Forestry, Hangzhou 310023, China; 15990054143@163.com

**Keywords:** cornstalk rind, H_2_O_2_/HAc delignification, cellulose aerogel, PVA, oil adsorption, thermal insulation

## Abstract

Cellulose-based aerogel has attracted considerable attention for its excellent adsorption capacity, biodegradability, and renewability. However, it is considered eco-unfriendly due to defibrillation of agriculture waste and requires harmful/expensive chemical agents. In this study, cornstalk rind-based aerogel was obtained via the following steps: green H_2_O_2_/HAc delignification of cornstalk rind to obtain cellulose fibers, binding with carboxymethyl cellulose (CMC)/polyvinyl alcohol (PVA) and freeze-drying treatment, and hydrophobic modification with stearic acid. The obtained aerogel showed high compressive strength (200 KPa), which is apparently higher (about 32 kPa) than NaClO-delignified cornstalk-based cellulose/PVA aerogel. Characterization of the obtained aerogel through SEM, water contact angle, etc., showed high porosity (95%), low density (0.0198 g/cm^−3^), and hydrophobicity (water contact angle, 159°), resulting in excellent n-hexane adsorption capacity (35 g/g), higher (about 29.5 g/g) than NaClO-delignified cornstalk-based cellulose/PVA aerogel. The adsorbed oil was recovered by the extrusion method, and the aerogel showed excellent recyclability in oil adsorption.

## 1. Introduction

As a globally important food crop, corn requires about four months to grow and generates 0.25 billion tons of cornstalks (CSs) in China annually [1,2,3]. However, less than half of these CSs are used for animal feed, and most are incinerated or thrown away as waste, causing serious environmental pollution. Corn plants consist of the cob, leaf, husk, and stalk. Corn husks can be used for bioplastic [1], textile fibers [3,4], cellulose nanocrystals [5], etc., due to their high cellulose content [6]. Scientific progress has created opportunities for cornstalk use in bioethanol, dissolved pulps [7], nanocellulose [8,9,10], cellulose fibers [11,12,13], polymer composites [6,14,15], bioplastics [16,17,18], aerogel [19], etc. Exploiting CSs efficiently would help recycle and add value to agricultural waste.

Cornstalk-based aerogel has attracted considerable attention for its excellent adsorption capacity, biodegradability, and renewability [20,21,22,23,24]. Wang et al. exploited cornstalk as raw materials to synthesize cornstalk-based carbon microsphere/reduced graphene oxide composite hydrogels for supercapacitors [20]. Xiong et al. used cornstalk to prepare CuS@cornstalk/chitin composite hydrogel for photodegradation and antibacterial mechanisms [21]. Li et al. obtained whole-component cornstalk aerogel using LiBr to treat cornstalk [22]. Lei et al. further enhanced the adsorption capacity of cornstalk-based hydrogel by crosslinking water-soluble polysaccharides and cornstalk cellulose [23]. Chen et al. used NaOH and NaClO to successfully treat cornstalk and obtain cellulose fibers with polyvinyl alcohol (PVA) as a binder for preparing superhydrophobic and superoleophilic aerogels with excellent adsorption capacity [24]. In all cases, NaClO, NaClO_2_, or LiBr were exploited to obtain or dissolve cellulose. However, the above chemicals are not eco-friendly since HClO, Cl_2_, or NaClO_3_ can be formed during the delignification process. In addition, treatment with NaClO_2_ must be carried out in an acetic acid solution at 80 °C and considerable amounts of toxic chlorinated compounds can easily be formed during the process [25,26]. The process for LiBr [22] must be carried out at a high temperature (150 °C), which greatly increases energy consumption. Moreover, the comprehensive stress of PVA-binding cellulose aerogel is relatively low (about 32 kPa) [24]. Therefore, synthesizing strong PVA-binding cornstalk cellulose-based aerogel via green processes is still challenging.

In this study, an alternative green method for synthesizing cornstalk rind cellulose-based aerogel with PVA binding is proposed. H_2_O_2_-HAc aqueous solutions were used to treat cornstalk rind and obtain cellulose fibers, subsequently binding with PVA to form a hydrogel. Finally, a cornstalk rind-based aerogel was fabricated via freeze-dry treatment of the above hydrogel. Hydrophobic modifications with stearic acid provide aerogels selective oil adsorption abilities. Fourier-infrared spectroscopy (FTIR), scanning electron microscopy (SEM), and X-ray diffractometry (XRD) were used to analyze the structure and composition of cornstalk rind, cornstalk rind-based cellulose fibers, and the obtained aerogel. The cornstalk-based aerogel’s properties were analyzed using contact angle meters, a universal mechanical testing machine, and a thermogravimetric analyzer (TGA). Its compressive strength was 200 kPa, which is apparently higher (about 32 kPa) than NaClO-delignified cornstalk-based aerogel using PVA as a binder. The adsorption performance of the aerogel was determined by measuring the adsorption amounts of n-hexane and linseed oil.

## 2. Experimental

### 2.1. Materials and Methods

Polyvinyl alcohol (PVA) (molecular weight 75,000; hydrolysis rate over 99%) was purchased from McLean Biochemical Co., (Shanghai, China). Corn stalk stalks (CSs) are provided by local farmers from Tieling, China. Carboxymethyl cellulose (CMC) (C4888, medium viscosity) was purchased from Sigma-Aldrich (Shanghai, China). Hydrochloric acid was provided by Fisher Scientific (Guangdong, China). The chemicals used for lignin removal were hydrogen peroxide (>30%), glacial acetic acid, and stearic acid, which were purchased from Jinshan Chemical Reagent Co., (Chengdu, China). Sodium hydroxide was supplied by Sci-tech (Guangdong, China).

### 2.2. Preparation of Rind Cornstalk Cellulose Microfibers (CSCF)

The cornstalk was peeled off to obtain cornstalk rind and crushed in a grinder. Then, a 15 *v*/*v*% H_2_O_2_/HAc solution synthesized by 30% hydrogen peroxide and glacial acetic acid at a molar ratio of 1:1 was used to delignify cornstalk rind at room temperature for 8 h [27,28] and washed with deionized water multiple times to remove the compounds and isolate parenchyma cells from long cellulose fibers. The treated cornstalk rind-based cellulose fibers were dried at room temperature for 12 h until the moisture content was less than 5 wt%.

### 2.3. Preparation of CMC/CSCF/PVA Aerogels

PVA was added to 100 mL of deionized water and stirred overnight at room temperature to completely dissolve the PVA and obtain PVA aqueous solutions (5% *w*/*v*). CMC aqueous solutions were prepared by dissolving CMC in HCl under magnetic agitation. CSCF composite aerogels with different PVA mass ratios were prepared by a single reactor method. CSCF and PVA aqueous solutions were added to the aforementioned CMC aqueous solutions. Weight ratios of 3/1 and 1/1 were predetermined for CSCF and CMC. After ultrasonic treatment, the above suspension was transferred to a refrigerator (−20 °C) overnight, and taken out to be thawed at room temperature. Freeze–thaw treatment was performed four times. The gel was removed, vacuum filtered, washed with deionized water, and finally freeze-dried into different solid aerogels for further characterization, where the mass percentages of PVA and solvent were 0.15% and 0.25%, respectively.

### 2.4. Hydrophobic Modification of CSCF/PVA Aerogels

Hydrophobic modifications of CSCF/PVA aerogels were carried out by dip-coating them with stearic acid–chloride (SAC). The aerogel was immersed in 30 mL of 0.06 mol stearic acid–chloride acetonitrile solution and reacted at 50 °C with triethylamine as an acid scavenger. The modified aerogel was thoroughly cleaned with n-hexane and freeze-dried. To further ensure solvent removal, the modified aerogel was placed in a vacuum oven at 50°C overnight. The obtained modified aerogel was referred to as CSCF/PVA-SAC aerogel, which was simplified to modified CSCF/PVA for subsequent discussion. Four aerogels were prepared by changing the mass fraction of CSCF, CMC, and PVA, as shown in Table 1.

### 2.5. Characterization

Morphology was determined using a Vega 3 scanning electron microscope (SEM) (Tescan, Brno, Czech Republic) at 5 KV voltage. To improve image quality, the sample was coated with gold using a Hummer X magnetron sputtering machine (Anatech, Brno, Czech Republic) before imaging. The mechanical performance of the samples was determined using a ProLine Z020TN universal testing machine (Zwick, Ulm, Germany). The amounts of cellulose, hemicellulose, and lignin in the samples were measured using the standard methods set out by the Technical Association of the Pulp and Paper Industry (TAPPI) [29,30,31,32]. Fourier-transform infrared (FTIR) spectra were recorded using an IR Tensor 100 FTIR spectrometer (Shimadzu, Tokyo, Japan). The mechanical properties of the samples were measured using a universal testing machine (CMT5504, MTS, Shanghai, China) with a tensile speed of 5 mm min^−1^ 32 was also used. Qualitative measurements were obtained of insulation properties under conductive heat sources. The upper surface temperature of the samples was measured with infrared thermography (UNI-T, UTi120s, UNI-T, Dongguan, China). The adsorption capacity of the material for n-hexane and rapeseed oil and the cycling ability of n-hexane were tested. A Bruker D8 X-ray diffractometer (MTS, Shanghai, China) with Cu Kα radiation (λ = 1.5418 Å) in the 2θ range of 4–50° was used for crystal structure characterization.

## 3. Results and Discussion

**Figure 1:**
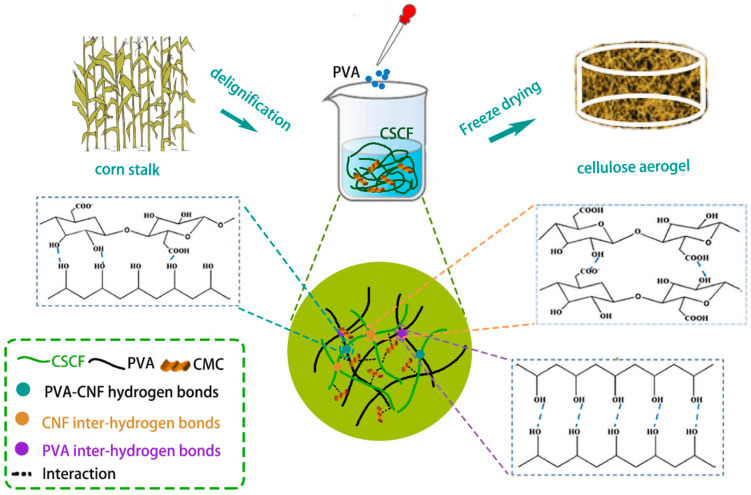
Schematic diagram of the synthesis of CSCFS/PVA aerogels and potential crosslinking interactions in aerogel networks.

Cornstalks (CSs) are usually exploited separately based on their heterogeneous structures of pith and rind. The central part of the pith is clearly distinguished by its low density, softness, and white color from the surrounding hard, dense, and light-brown fibrous rind [8]. The pith mainly consists of parenchyma cells with weak mechanical properties. As for the CSs rind, it mainly consists of fiber bundles surrounded by parenchymal cells [8,13,22]. As shown in Figure 1, the CSs rind powder was used as a raw material and delignified with H_2_O_2_/HAc to obtain cellulose fibers. The obtained cornstalk cellulose fibers (CSCF) and CMC were dispersed in PVA aqueous solutions to form suspensions, which were then poured into a mold and frozen. Afterward, a CSCFS-based aerogel was obtained by freeze-drying. Finally, hydrophobic modifications were carried out by immersing the above aerogels in stearic acid solutions (Figure 1).

### 3.1. Preparation of Cornstalk-Based Aerogel

**Figure 2:**
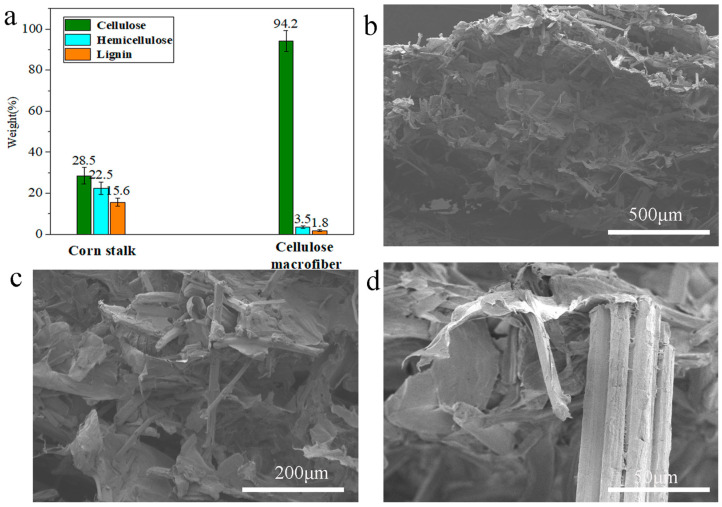
(**a**) Cellulose, hemicellulose, and lignin content in cornstalk rind before (left) and after (right) delignification. Five samples were measured in each sample, and the test results were recorded as an average. (**b**–**d**) Cross-sectional SEM images of CMC/CSCFS/PVA aerogels.

CSCFS was obtained via H_2_O_2_/HAc delignification of crushed cornstalk rind. Figure 2a shows that the cellulose content increased from 28.5% to 94.2% after delignification. Cellulose fibers (size and length are about ten micrometers and hundred micrometers, respectively) were generated via delignification. Then, the obtained cellulose fibers and CMC were dispersed in a PVA aqueous solution to form suspensions that were further converted to cornstalk cellulose-based aerogel via freeze-thaw treatment. The porous structures of cornstalk cellulose-based aerogels were characterized by SEM. As shown in Figure 2b, the obtained aerogel showed a 3D porous structure consisting of 1D cornstalk cellulose fibers, CMC microparticles, and PVA film. Cornstalk cellulose-based aerogel shows porous structures with “fiber/particle/flake entanglements”. PVA films appear to coat the surface of the cellulose fibers. Notably, the ends of fibers connected to the PVA flake form a 3D porous structure. Specifically, PVA flakes functioned as a binder, while both cornstalk cellulose fibers and CMC particles served as aerogel skeletons and contributed to high tensile and compressive strength.

### 3.2. XRD and FTIR of Characterizations

**Figure 3:**
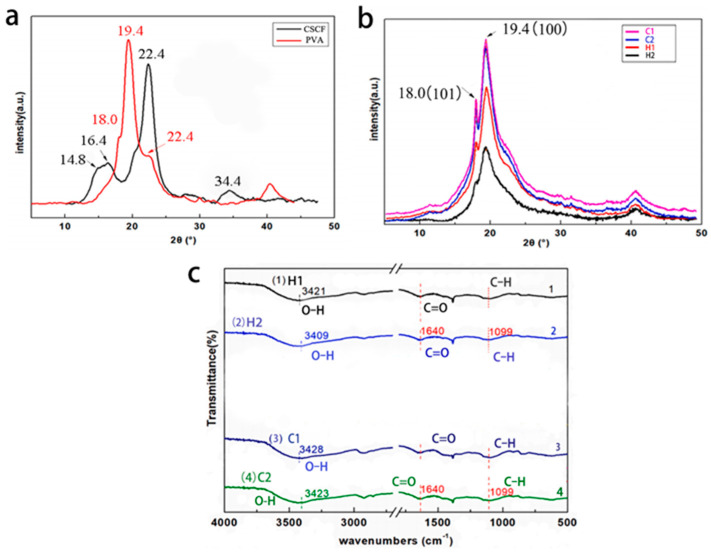
XRD patterns and FTIR of CSCFS, PVA, and aerogel samples. (**a**) XRD patterns of pure CSCFS and PVA. (**b**) XRD patterns of aerogels with different components (y = H1, H2, C1, C2). (**c**) FTIR spectrum of aerogels with increased CSCFS (lines 1 and 2) and PVA contents (lines 3 and 4).

The crystalline structures of CSCFS, PVA, and the obtained aerogel were characterized by XRD. The XRD pattern of pure PVA had a strong peak at 2θ = 19.4° (101) and two weak shoulder peaks at 2θ = 18.0° (100) and 22.4° (200) [33]. The typical peak of CSCFS cellulose is located at 2θ = 22.4° (200), and there were two weak diffraction peaks at 2θ = 16.4° (110) and 34.4° (004) (Figure 3a) [3,34]. With increasing CSCFS content, the plane intensity (200) is enhanced (Figure 3b). At the same time, with the increase in PVA and CSCFS concentration, the crystallinity increased from 25% to 45%, which can be measured by peak intensity. These results are consistent with existing data, indicating that PVA prefers to form hydrogen bonds with CSCFS cellulose [35]. FTIR spectra (Figure 3c) were also used to characterize functional groups of samples. All the prepared aerogels have broad and strong peaks between 3300 and 3650 cm^−1^, resulting from intermolecular and intramolecular hydrogen bonding between PVA and CSCFS. The peak shifts to a lower wavenumber when the CSCFS content increases. Similarly, with the increase in PVA concentration, the peak at 3400 cm^−1^ shifted to a lower wavenumber. Characteristic peaks of carbonyl stretching at 1164 cm^−1^ in protonated CMC and C-O stretching at 1099 cm^−1^ in cellulose were observed and increased with cellulose content. The results show that an increase in CSCFS enhances hydrogen bond formation in aerogels. Therefore, PVA and CSCFS content affect intermolecular and intramolecular interactions. In other words, the higher the PVA and CSCFS content, the stronger the interaction between them, which determines the porous structure, physicochemical properties of the polymer, and the long-term stability of aerogels.

### 3.3. Mechanical Properties of Aerogel

**Figure 4:**
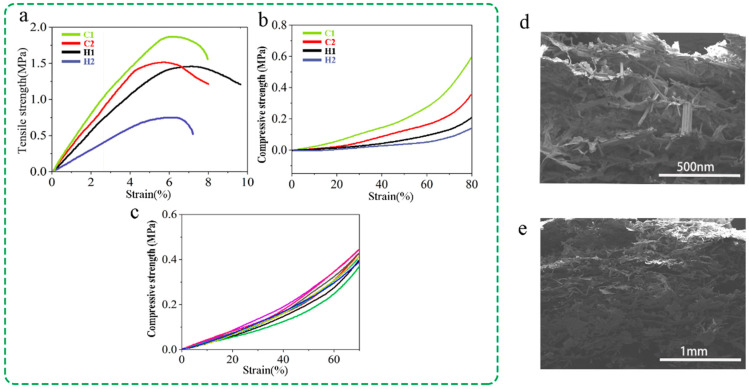
Strain–stress curves of four aerogels. (**a**) Tensile strength of the aerogel at various concentrations; (**b**) compressive strength of aerogels; (**c**) strain–stress of C1 under loading–unloading cycles. SEM images of aerogel (**d**) before and (**e**) after compression.

As shown in Figure 4a, the tensile strength increases with the increase in CSCFS and PVA content in aerogels. The tensile strength of H2 aerogel is 0.71 MPa. When the mass fraction of CSCFS increases to 75% and the PVA content is 0.25%, the tensile strength increases to 1.8 MPa. The compressive stress increases from about tens of kPa to about 200 kPa with the increase in PVA concentration (Figure 4b), which is apparently higher (about 32 kPa) than NaClO-delignified cornstalk-based aerogel using PVA as a binder [24]. According to these findings, the higher the PVA content, the more microcrystalline domain crosslinking sites form in the unit space; that is, the higher the PVA content, the denser the aerogel structure. In addition, considering that cellulose’s mechanical properties are higher than lignin [36], independently of moisture and environmental conditions, the residual lignin of CSCFS is lower than NaClO-delignified cornstalk cellulose fibers, which also have higher aerogel mechanical properties. Aerogel’s mechanical cycling performance was tested on a universal testing machine, which determined the compressive stress–strain relationship for the first and tenth cycles. Figure 4c shows a strain–stress curve of 7 h under a continuous loading–unloading cycle. The strain–stress curves of the first to tenth cycles basically coincide, indicating that CSCFS/PVA aerogel has good self-recovery ability under strain. During the compression process, the aerogel’s ability to withstand compressive stress decreases with the increase in cycles. Therefore, when the strain reaches 60%, the rising curve does not overlap and the stress drop is not significant. This study is expected to obtain cellulose aerogels with cellulose and carboxymethyl cellulose as the “skeleton”. Cellulose is connected by hydrogen bonds, resulting in regular pores between the “skeletons” of cellulose microfibers, leading to the excellent compressive elastic properties described above. SEM images (Figure 4d,e) of aerogels before and after the compression process show minimal damage to the lamellar structure.

### 3.4. Thermogravimetric Analysis

**Figure 5:**
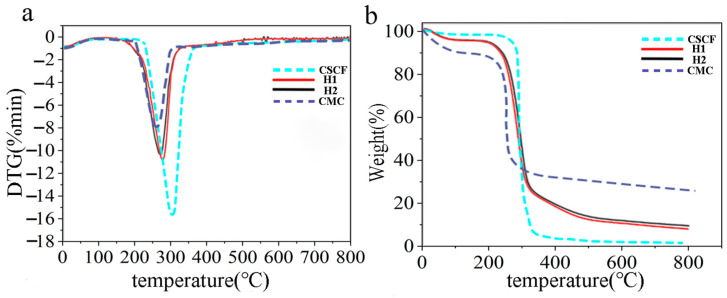
(**a**) DTG and (**b**) TGA curves of CSCFS, CMC (dash lines), aerogels of C1 and C2 (solid lines).

Thermogravimetric analysis of CSCFS, CMC, and aerogels was performed, as shown in Figure 5. Compared to pure CMC, CSCFS has a higher initial thermal decomposition temperature (T_onser_), and the main derivative thermal degradation (DTG) peak is located at a higher temperature. The decrease in CMC’s thermal stability is attributed to the carboxylic acid portion, which has been reported elsewhere [37]. At 257 °C, the DTG peaks of different foams were passivated, while the DTG peaks of C1 and C2 shifted to the high-temperature region (Figure 5a). Compared to CMC, the thermal stability of C1 and C2 aerogels was significantly improved (about 54.9°) due to CSCFS’s high thermal stability. However, the experimental values of T_onser_ and T_p_ are higher than the theoretical values, which may be because the cellulose content of C1 and C2 is higher than that of CMC (Figure 5b). The CSCFS/PVA gel material has excellent thermal stability, comparable to most existing cellulose-based and CMC-based materials [37]. These results indicate that the improved thermal stability cannot be solely attributed to cellulose’s high thermal stability, but rather to the possible existence of specific interactions between CSCFS/CMC and PVA.

### 3.5. Adsorption and Recycling Capacity of CMC/CSCFS/PVA Aerogels

**Figure 6:**
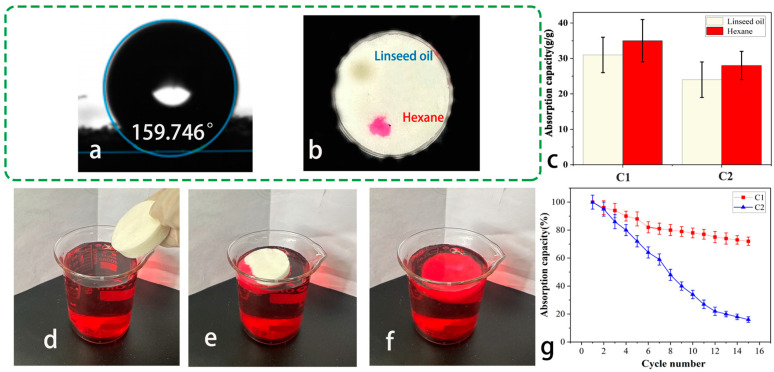
Reusability of CSCFS/PVA aerogels for n-hexane. (**a**) The water contact angle of CSCFS/PVA aerogel; (**b**) absorption of n-hexane (red) and linseed oil (colorless); (**c**) absorption capacity of aerogels to hexane and linseed oil; (**d**) recovered CSCFS/PVA aerogels; (**e**,**f**) The absorption behavior of CMC/CSCFS/PVA aerogels; (**g**) the absorption capacity percentage of CMC/CSCFS/PVA aerogels during 15 cycles.

After hydrophobic modification, the water contact angle of stearic acid-modified CMC/CSCFS/PVA aerogel was 159° (Figure 6a), indicating its superhydrophobicity [38]. The adsorption capacity of hexane and linseed oil droplets was tested. These droplets were quickly absorbed and entered the foam within 10 s (Figure 6b). Aerogels’ high affinity for non-polar solvents can be attributed to the capillary force promoted by their porous structure. The high affinity of the aerogels for non-polar solvents can be attributed to the capillary force promoted by their porous structure. It should be noted that the viscosity and density of the liquid also affect the adsorption performance of the aerogel [39]. In the case of oil adsorption, the higher the viscosity, the better the adsorption performance. In the case of organic solvent adsorption, the higher the density, the better the adsorption performance. Figure 6c shows the absorption capacity of CSCFS/PVA aerogels to n-hexane and linseed oil. Notably, the adsorption capacity of our aerogel to n-hexane is 35 g/g, which is higher (about 29.53 g/g) than NaClO-delignified cornstalk-based aerogel using PVA as a binder [24]. A simple extrusion method was exploited to adsorb and extract oil using CSCFS/PVA aerogels. The adsorption and recycling performance of the CMC/CSCFS/PVA aerogel for n-hexane (diesel dyeing) is shown in Figure 6d–g. The aerogel is saturated with hexane within a few seconds (Figure 6a–c). Both C1 and C2 aerogels can be reused. Due to the toughness of PVA, CSCFS/PVA aerogels are elastic and can be reused at least 15 times with high absorption efficiency [40]. Compared to aerogels with low PVA content, CSCFS/PVA aerogels with higher PVA content showed better recoverability. As shown in Figure 6d, the n-hexane absorption percentage of CSCFS/PVA aerogel decreased from 100% to 75% after 15 cycles.

### 3.6. Thermal Insulation Performance of CSCFS/PVA Aerogels

**Figure 7:**
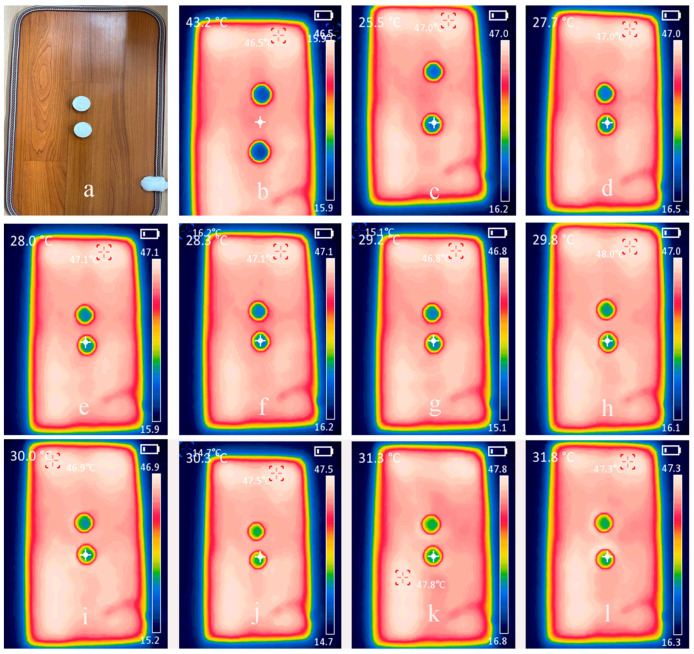
(**a**) Digital images of aerogels C1 (**top**) and C2 (**down**). (**b**) The thermal images of the aerogels before the microwave test. (**c**–**l**) Thermal images were captured after irradiating neat C1 and C2 aerogels above a microwave oven (250 W) for 10 s. Images were captured at 10 s time intervals.

In our daily lives and buildings, most work using thermal insulation materials is carried out at relatively low temperatures (below 45 °C). To study the heat absorption properties of PVA/CNF aerogels, we placed PVA/CNF aerogels in a heating device (250 W) for experiments. Figure 7 shows the thermal images of the original C1 and C2 aerogels before microwave exposure. After the sample was exposed to a thermal radiation pad for 8 s, the two aerogels remained at about 27 °C due to aerogels’ high porosity. The infrared thermal images of the two aerogels were recorded every 10 s (see Figure 7). According to Newton’s law of cooling, the heat loss rate of the system is determined by the following equation:

$$\frac{dQ}{dt}=hA\left(T_{S}-T_{\infty }\right).$$

where, *Q* is heat energy, *h* is the heat transfer coefficient, *A* is the surface area, *T_S_* is the surface temperature, and *T_∞_* is ambient temperature. As shown in Figure 7, the CMC/CSCFS/PVA aerogel temperature increases proportionally after 6 s of thermal radiation. The CSCFS/PVA density is 0.0198 g/cm^3^. After measuring, the thermal conductivity of the sample was 0.053 W m^−1^ K^−1^. Due to the low thermal conductivity of air, heat energy was radiated into the surrounding air bag through convective heat transfer. In addition, it was found from TGA (Figure 6) that the CSCFS/PVA aerogel was stable at 200 °C without obvious degradation, indicating that our aerogel was suitable for use at higher temperatures.

## 4. Conclusions

Cornstalk-based cellulose fiber prepared by the H_2_O_2_/HAc delignification method combined with CMC served as the 1D/0D skeleton with PVA binding to form CMC/CSCFS/PVA aerogel. Due to the synergistic mechanisms of CMC and CSCFS, the obtained aerogels showed low density (0.0198 g/cm^−3^), hydrophobicity (water contact angle, 159°), and high compressive strength (about 200 kPa), which is apparently higher (about 32 kPa) than NaClO-delignified cornstalk-based aerogel using PVA as a binder. Owing to their 3D porous structure and hydrophobicity (water contact angle, 159°), our aerogels have high oil adsorption capacity (35 g/g) and excellent thermal insulation. Notably, our aerogel’s adsorption capacity to n-hexane is higher (about 29.53 g/g) than NaClO-delignified cornstalk-based aerogel using PVA as a binder. The adsorption capacity of the aerogel after repeated use (15 times) showed that the aerogel has better recyclability and reusability than the previous straw fiber aerogel for hexane recovery. Our green strong CSCFS/CMC/PVA aerogel provides a competitive alternative to adsorbent materials and thermal insulation materials, adding value to agriculture waste. Based on our CMC/CSCFS/PVA aerogel, the next generation of filter and coating materials can be designed and manufactured to capture oil-based solvents in future research routes. 

## Figures and Tables

**Table 1:** Components of the four aerogel samples.

	H1	H2	C1	C2	Quality
CMC	1.5	1.5	1.5	1.5	g
CSCF	1.5	4.5	4.5	4.5	g
PVA	0.125	0.125	0.125	0.25	g

## Data Availability

Data are contained within the article.
